# Metastasis of endometrial cancer to right hemidiaphragm: A case report

**DOI:** 10.1016/j.ijscr.2019.06.028

**Published:** 2019-06-20

**Authors:** Ivan Sergeevich Gruzdev, Ivan Andreevich Blokhin, Aleksey Nikolaevich Lednev, Aleksey Aleksandrovich Pechetov, Maksim Aleksandrovich Makov, Yuriy Sergeevich Esakov, Artem Garrievich Arevin, Andrey Vyacheslavovich Glotov, Grigory Grigorievich Kаrmаzаnovsky

**Affiliations:** a A.V. Vishnevsky National Medical Research Centre of Surgery, Bolshaya Serpukhovskaya str. 27, 117997, Moscow, Russia; b Research and Practical Clinical Centre of Diagnostics and Telemedicine Technologies, st. Srednyaya Kalitnikovskaya str. 28, 109029, Moscow, Russia; c Department of Thoracic Surgery, A.V. Vishnevsky National Medical Research Centre of surgery, Bolshaya Serpukhovskaya str. 27, 117997, Moscow, Russia; d V. Vishnevsky National Medical Research Centre of surgery, Pirogov Russian National Research Medical University, Bolshaya Serpukhovskaya str. 27, 117997, Moscow, Russia

**Keywords:** Metastasis to the diaphragm, Endometrial cancer, CT, Diaphragmotomy

## Abstract

**Introduction:**

Tumors of the diaphragm are uncommon. The overwhelming number of cases is metastatic combined with metastases to the liver, lungs and other organs. Only a minority of cases are described as solitary lesions.

**Case presentation:**

Fifty-five years old female with a history of radical curative surgery for pT3N0M0 endometrial cancer eight years ago was admitted to the Department of Thoracic Surgery with a feeling of discomfort in the right hypochondrium. The contrast-enhanced MDCT revealed a large, well-circumscribed lesion of the right hemidiaphragm deforming upper contour of the liver. A clear boundary between the lesion and the liver suggested former’s diaphragmatic origin. PET-CT did not show any distant metastasis. Intraoperative revision revealed a tumor growing from the dome of the diaphragm with well-defined contours and without any signs of lung involvement. The diaphragmotomy was performed. The morphological study with immunohistochemistry showed an endometrial carcinoma metastasis to the diaphragm.

**Conclusion:**

The diaphragm lesions can have various etiology, but a probability of tumor metastasis after a previous radical surgery should not be excluded. Preoperative differential diagnostics can be difficult, leaving surgical treatment followed by a pathology study as the most informative diagnostic method of tumor morphology.

## Highlights

• Metastatic involvement of the diaphragm is more common than primary malignancy.
• Isolated diaphragmatic metastasis is very rare.
• Contrast-enhanced multidetector computed tomography provides essential information for preoperative planning.
• A pathology study and surgical revision are the most informative diagnostic methods.

## 1 Introduction

Tumors of the diaphragm are uncommon with an overwhelming number of cases being metastatic. Secondary malignant tumors of the diaphragm are most often combined with metastases to liver, lungs and other organs [1]. Only a small number of solitary secondary diaphragmatic lesions is reported in the literature [2,3]. We present a clinical case of metastatic lesion of the right hemidiaphragm in a patient with endometrial adenocarcinoma eight years after curative surgery. This case report is compliant with the SCARE criteria [4].

## 2 Clinical case

**Fig. 1:**
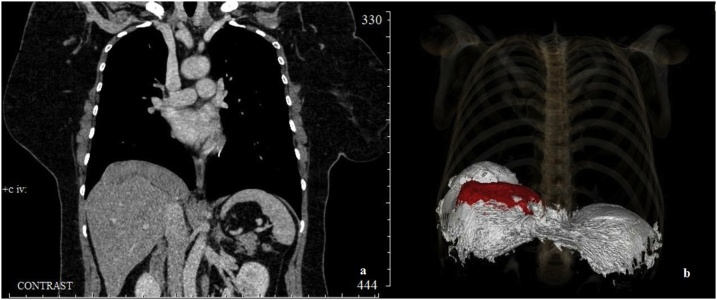
Chest CT coronal scan with 3D CT volume rendering picture of the tumor and the diaphragm.

Fifty-five years old female was admitted to the Department of Thoracic Surgery in July 2018 with heaviness in the right hypochondrium. In 2010, she underwent radical curative surgery for pT3N0M0 endometrial cancer (hysterosalpingooophorectomy and greater omentum resection). Histological examination depicted an endometrial glandular squamous carcinoma with invasion of the uterine myometrium. The adjuvant combined radiation and chemotherapy was as follows: 40 Gy for pelvis, 20 Gy for vaginal stump and three courses of cyclophosphamide, adriamycin, 5-fluorouracil (CAF scheme). Annual check-ups have not shown any signs of relapse for seven years. In May 2018, the patient noted a new dull, nagging pain in the right hypochondrium. Abdominal ultrasound revealed a 22 × 56 mm focal liver lesion in the SVIII. Contrast-enhanced MDCT of the chest and abdomen was performed to clarify lesion’s nature, location, and size. The study revealed a large, well-circumscribed tumor in the SVIII of the liver. Positron emission tomography combined with computed tomography was used to determine the lesion’s metabolic activity and exclude distant metastases. The scan visualized a large, hypermetabolic 11.4 × 13.5 × 10.7 cm lesion of the right hemidiaphragm. We hypothesized that the tumor originates from the diaphragm and invades the right liver lobe, deforming its upper contour in a wavy manner. Computed tomography suggested a clear the boundary between the diaphragmatic tumor and liver in the form of the fatty tissue layer. Post-processing with volume rendering depicted this relationship more vividly (Fig. 1).

**Fig. 2:**
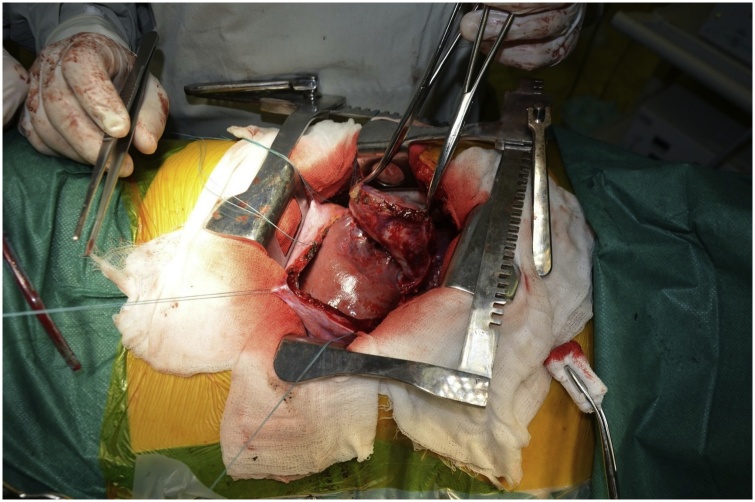
Intraoperative picture: tumor is closely adjacent and connected with the right liver lobe (SVII, SVIII) by loose adhesions without signs of invasion.

**Fig. 3:**
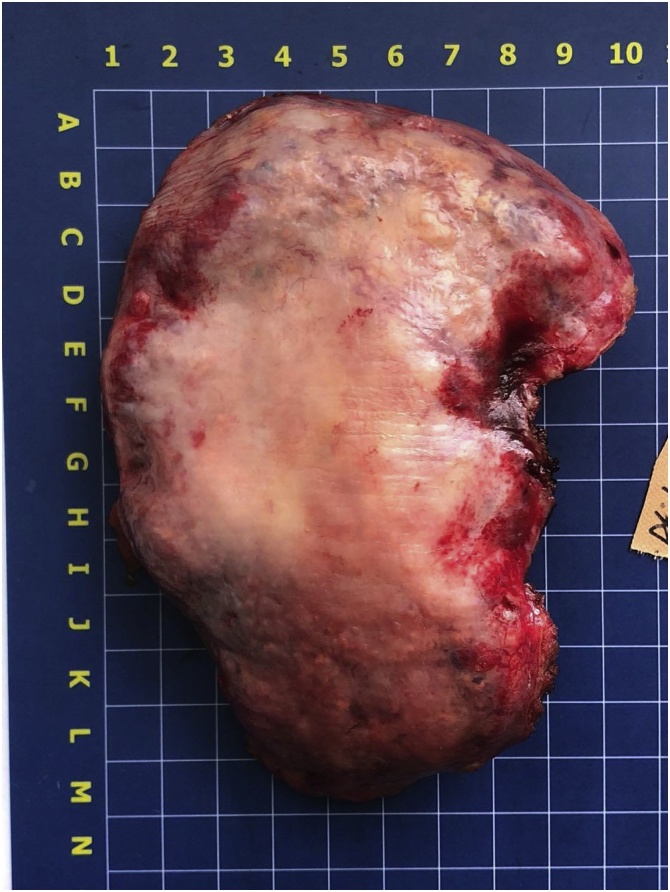
The gross specimen.

**Fig. 4:**
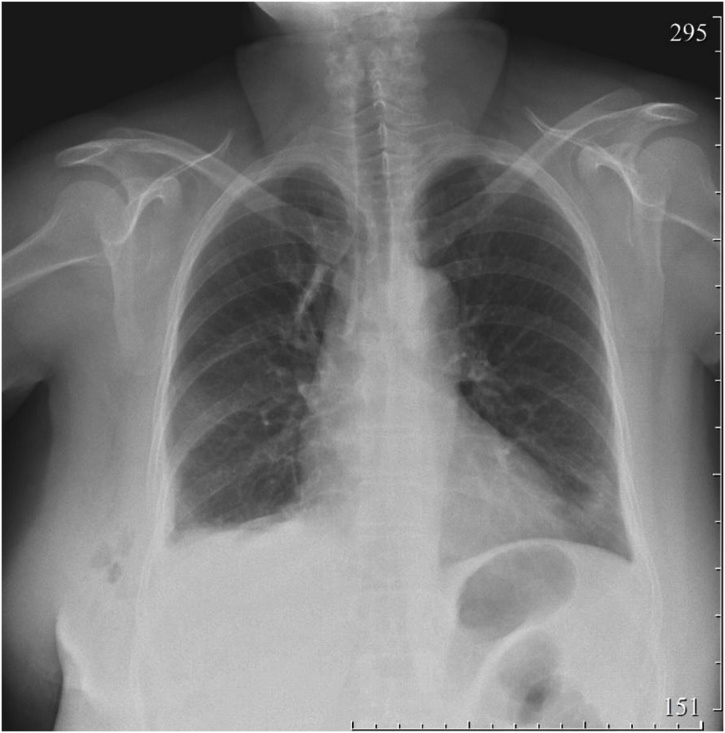
Afteroperative chest X-ray: formed right diaphragmatic dome at the seventh intercostal space.

Given the lesion’s size, solitary nature, close contact with the right liver lobe, clear tumor margins without lung involvement, the multidisciplinary team decided to treat the case surgically. The team initiated the video-assisted thoracoscopic surgery procedure with subsequent conversion to the open right lateral thoracotomy through the fifth intercostal space due to lesion’s size. Intraoperative revision revealed a tumor growing from the dome of the diaphragm with clear even contours and without any signs of lung involvement. The diaphragmotomy was performed: the tumor is closely adjacent and connected with the right liver lobe (SVII, SVIII) by loose adhesions without signs of invasion (Fig. 2). The tumor was separated from the right liver lobe by sharp dissection. The right diaphragmatic dome resection along with the neoplasm had the minimal resection margin of one centimeter. The gross specimen measured 9.5 × 12.5 cm and was forwarded to the pathologist (Fig. 3). We sutured the diaphragmatic defect with local tissues (separate U-shaped seams without tension). The operation concluded by forming right diaphragmatic dome at the seventh intercostal space (Fig. 4).

The postoperative period was unremarkable. Physiotherapeutic inhalations with antiseptic solutions were performed to prevent postoperative complications. The pleural drainage was removed, and the patient mobilized on the second day. The follow-up chest radiograph was within the normal limits, right hemidiaphragm located at the seventh intercostal space. Therefore, the patient was discharged on the seventh-day post-operation. On the follow-up chest and abdominal contrast-enhanced CT at six months after the operation, there were no signs of recurrence.

The morphological study showed that the tumor had been an endometrial carcinoma metastasis to the diaphragm.

**Fig. 5:**
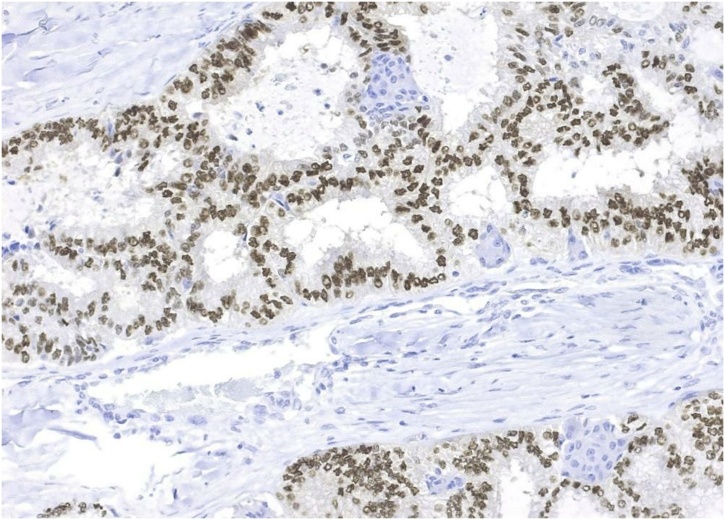
Immunohistochemical study (diaminobenzidine, hematoxylin), x200 magnification. Tumor cells express the progesterone receptor (Y85 clone, Cell Marque).

Immunohistochemistry was as follows:

Estrogen receptor (clone SP1, Cell Marque) - IS +++, 3 points; PS 40%, 4 points; TS = 7 points; Progesterone receptor (clone Y85, Cell Marque) - IS +++, 3 points; PS 40%, 4 points; TS = 7 points (Fig. 5).

## 3 Discussion

M. Grancher first described a benign diaphragmatic tumor in 1868 at an autopsy [5]. Primary diaphragmatic tumors are predominantly benign (fibromas, lipomas) [6]. Primary malignant or metastatic tumors of the diaphragm are rare with only a handful of published case reports. Until 2017, less than 200 clinical observations were available with the mesenchymal tumors being most common [[7], [8], [9]]. Metastatic involvement of the diaphragm is more common than primary malignancy. The epidemiological data on the prevalence of primary and secondary malignant diaphragmatic lesions are lacking. However, lung, liver and ovarian cancer metastases have been described [2,3,10].

In 2012, 527 600 new cases of endometrial cancer were diagnosed worldwide [11]. Mortality ranged from 1.7 to 2.4 per 100,000 women. In developed countries, endometrial cancer is the most common gynecological malignancy with more than 60 000 new cases and more than 10 000 deaths per annum [12]. The metastases to pelvic organs, para-aortic, intra-abdominal and intrathoracic lymph nodes, peritoneum, and lungs are the most commons. The involvement of parenchymal organs (liver, adrenal glands, spleen, brain), bones is relatively rare [13]. We could not find publications on isolated diaphragmatic metastasis to the diaphragm in primary endometrial cancer, but there are case reports on such metastases in ovarian cancer [10,14]. According to Eisenkop S. et al., the diaphragm is affected in up to 40% of patients with metastatic ovarian cancer, but the involvement is usually diffuse contraindicating any surgical treatment. Our clinical report describes the treatment of solitary metastasis to the right hemidiaphragm diagnosed eight years after the radical treatment of uterine endometrial adenocarcinoma.

From a clinical point of view, the diagnostics and treatment of diaphragmatic tumors are of great interest. Due to the casuistic nature of such lesions, there were no recommendations on preoperative verification and staging of diaphragmatic tumors at the time of writing. In the presented clinical observation, the metastasis manifested eight years after supposedly curative surgery. Despite the medical history and presence of the feeding vessel on computed tomography, it was impossible to exclude a primary malignancy before the operation. We decided to operate the lesion after considering its size, location, growth pattern and the high risk of complications associated with a transthoracic biopsy.

## 4 Conclusion

The type of surgical treatment depends on the size and nature of the neoplasm. Plastics with the autogenous tissue or mesh implants after benign tumor resection provides the best results. In the case of an indeterminate diaphragmatic lesion, follow-up may be advisable. Malignant lesions of the diaphragm comprise a separate group. Preoperative differential diagnostics in an isolated tumor can be challenging, leaving surgical treatment followed by a pathology study as the most informative diagnostic method.

## Conflicts of interest

None.

## Funding

The case report was written without any funding.

## Ethical approval

This is a case report without personal data or personal figures of the patient. Therefore no ethical approval is required.

## Consent

Written informed consent was obtained from the patient for publication of this case report and accompanying images. A copy of the written consent is available for review by the Editor-in-Chief of this journal on request.

## Author contribution

I.S. Gruzdev - data curation, writing - original draft, visualization, project administration.

I.A. Blokhin - data curation, writing - original draft, visualization.

A.N. Lednev - data curation, writing - original draft.

A.A. Pechetov - conceptualization, writing - review & editing.

Yu.S.  Esakov - writing - review & editing, data curation.

M.A. Makov - data curation.

A.G.  Arevin - data curation.

A.V. Glotov - data curation (morphological study & immunohistochemistry).

G.G. Karmazanovsky - conceptualization, writing - review & editing, project administration, supervision.

## Registration of research studies

The case report does not contain data of human studies.

## Guarantor

Grigory G. Kаrmаzаnovsky.

## Provenance and peer review

Not commissioned externally peer reviewed.
